# Encouraging appropriate gestational weight gain in high‐risk gravida: A randomized controlled trial

**DOI:** 10.1002/osp4.565

**Published:** 2021-09-22

**Authors:** Awathif Dhanya Mackeen, Amanda J. Young, Shawnee Lutcher, Vonda Hetherington, Jacob W. Mowery, Jennifer S. Savage, Danielle Symons Downs, Lisa Bailey‐Davis

**Affiliations:** ^1^ Department of Obstetrics and Gynecology Geisinger Danville Pennsylvania USA; ^2^ Department of Population Health Sciences Geisinger Danville Pennsylvania USA; ^3^ Biostatistics Core Geisinger Danville Pennsylvania USA; ^4^ Obesity Institute Geisinger Danville Pennsylvania USA; ^5^ Nutrition Services Geisinger Danville Pennsylvania USA; ^6^ Department of Nutritional Sciences The Pennsylvania State University University Park Pennsylvania USA; ^7^ Department of Kinesiology The Pennsylvania State University University Park Pennsylvania USA

**Keywords:** gestational weight gain, nutrition counseling, obesity, pregnancy

## Abstract

**Trial Design:**

Excessive gestational weight gain (GWG) can increase pregnancy morbidity and is particularly problematic for women with pregestational obesity. A lifestyle modification intervention was introduced to gravida with obesity to decrease excessive GWG as compared to usual care (UC).

**Methods:**

A randomized controlled trial was conducted to improve healthy lifestyle behaviors to manage appropriate GWG. Consenting participants with prepregnancy obesity and singletons ≤17 weeks were randomized to (1) **Usual Care (UC)**: usual written educational materials and counseling by obstetric provider or (2) **Enhanced Care (EC)**: UC plus (a) personalized letter from physician detailing appropriate GWG; (b) access to individualized GWG chart; (c) ongoing counseling with registered dietitian/nutritionist (RDN). The primary outcome was proportion with GWG ≤9.1 kg, as this is upper limit recommended by Institute of Medicine (IOM). Total GWG and GWG as less than/within/greater than IOM recommendations (in aggregate and stratified by obesity class), and pregnancy/neonatal outcomes were evaluated as secondary outcomes.

**Results:**

Analyses included 105 participants in EC and 109 in UC arms. The groups had similar demographics: 46% with class I obesity, 26% class II, and 28% class III. There were no group differences for any GWG, pregnancy, or neonatal outcomes when analyzed in aggregate. As compared to those randomized to the EC arm, participants in UC arm with class I obesity gained 1.4 kg less and those with class II obesity were significantly more likely to gain within IOM guidelines (14.8% vs. 40.0%, adjusted *p* = 0.04). Participants with class III obesity randomized to EC arm were more likely to gain within IOM guidelines as compared to participants randomized to UC arm (29.0% vs. 6.7%, adjusted *p* = 0.02).

**Conclusion:**

There were no differences in GWG observed between groups when analyzing participants in aggregate. However, a physician's letter detailing appropriate GWG, patient portal access to a personalized GWG chart, and RDN consultation were helpful for encouraging GWG within IOM guidelines for women with prepregnancy class III obesity. Women with class I or II obesity had better GWG outcomes without these additional interventions.

## 1 BACKGROUND

The increasing rate of maternal obesity provides a major challenge to obstetric practice. Compared to women with normal weight, women with obesity (prepregnancy BMI ≥30.0 kg/m^2^) and severe obesity (BMI ≥40.0 kg/m^2^) have a 45% and 88% increased number of admissions, respectively; this results in antenatal hospital costs that are 5‐fold that of women with normal weight.^1^, ^2^ These increased admissions are largely attributed to obesity‐related complications, including excessive gestational weight gain (GWG), preterm birth, preeclampsia, gestational diabetes, and cesarean delivery.^2^, ^3^, ^4^, ^5^ In fact, excess GWG is a stronger determinant of preterm birth and large for gestational age neonates than prepregnancy body mass index (BMI).^6^ Therefore, there are tremendous public health implications of maternal BMI and GWG^6^ and studies should assess perinatal health outcomes in addition to GWG.^7^

Over 50% of pregnant women with obesity gain more weight during pregnancy than the Institute of Medicine (IOM) recommends: appropriate GWG in pregnancy is 5.0–9.1 kg for gravida with obesity.^8^, ^9^ Although a majority of obstetricians report calculating BMI and using IOM guidelines to modify recommendations for GWG,^10^ studies have found that two thirds of women lack knowledge of GWG guidelines, and few receive adequate advice on controlling GWG.^11^, ^12^ In a Pennsylvania study, women indicated receiving a range of advice including advisement to gain too much weight, no recommendation for GWG at all, and a lack of concern from providers about excessive GWG.^13^ Importantly, patient experience in this arena is not optimal as women report anxiety about appropriate GWG, desire and value GWG advice from their providers, and perceive a lack of GWG counseling in prenatal care.^13^, ^14^, ^15^

The American College of Obstetrics and Gynecology recommends that women with obesity receive diet and exercise counseling guided by IOM recommendations for GWG during pregnancy.^16^ Obstetricians lack confidence in their ability to affect GWG and face challenges such as inadequate time, reimbursement, and training for provision of quality weight management care.^10^, ^17^ Current clinical guidelines suggest that providers involve a registered dietitian/nutritionist (RDN) in weight management care from preconception through the postpartum period, but providers report having inadequate referral resources.^17^ And while referrals from obstetricians to RDNs are common for gestational diabetes, only 3 out of 10 obstetricians refer patients to RDNs for weight management.^10^ These infrequent referrals for weight management may signal secular trends and a hesitation to recognize obesity as a disease.^18^ Several randomized controlled trials (RCTs) have demonstrated modest effects with diet and physical activity interventions in decreasing GWG,^19^, ^20^ particularly when education, feedback, and behavioral change techniques were used.^5^ This approach is consistent with the guidelines practiced by RDNs.^21^

Given that women with obesity expect GWG care, and that obstetric providers lack confidence in providing GWG care,^22^ innovative care models are needed,^23^ specifically those that increase access to skilled professionals such as RDNs.^24^ The aim of this study was to decrease excessive GWG among women with pregestational obesity. Usual obstetric care (control) was compared to enhanced care (EC) that included a personalized lifestyle modification intervention delivered by RDNs. The hypothesis was that the intervention would decrease excessive GWG among obese gravida, and therefore, improved maternal and neonatal outcomes.

## 2 METHODS/DESIGN

This RCT was implemented from 2016 to 2019. Participants sought prenatal care in 1 of 13 Obstetric and Maternal‐Fetal Medicine Geisinger outpatient offices located in 10 counties in predominately medically underserved areas within northeastern and central Pennsylvania. Eligible pregnant women had to (a) have a prepregnancy BMI ≥30.0 kg/m^2^; (b) be enrolled prior to 17 0/7 weeks gestation; (c) be pregnant with a singleton fetus; (d) have gained less than 5.0 kg between their prepregnancy weight and study enrollment; and (e) have access to a phone. Exclusions included planned delivery outside of Geisinger (and non‐Geisinger prenatal care providers), vegan diet, malabsorptive conditions/hyperemesis gravidarum, previous enrollment in this study in prior pregnancy, active diagnosis of cancer, acquired immunodeficiency syndrome, and current care by palliative medicine provider. This study was approved by the Geisinger Institutional Review Board and registered on ClinicalTrials.gov (NCT02963428).

### 2.1 Recruitment

A multipronged approach was used to recruit eligible patients who received care from participating offices. An informational study flyer was posted in all participating offices, and clinic staff provided potentially eligible participants with a copy of the study flyer at the scheduled visit. Women with scheduled prenatal appointments in participating offices who were considered potentially eligible based on available criteria pulled from the Geisinger's Epic^®^ electronic health record (EHR) were identified and offered inclusion, either in‐person or by follow‐up phone call.

### 2.2 Study flow and randomization

During screening (and prior to randomization), all potential participants answered a series of questions to ensure that all inclusion/exclusion criteria were met. After screening and completion of signed consent forms, participants were randomized into either the *Usual Care* (UC) or the *Enhanced Care* (EC) arm. Randomization was based on a 1:1 computer‐generated schema in random‐sized blocks (block size 2 or 4) stratified by obesity class (i.e., BMI 30.0–34.9 kg/m^2^ [class I], 35.0–39.9 kg/m^2^ [class II], ≥40.0 kg/m^2^ [class III]) and location of enrollment site (i.e., northeast or central Pennsylvania region). The randomization schema was created by the Geisinger Biostatistics Core and maintained via an electronic database.

Obstetric providers and RDNs received training on recommended guidelines for GWG; RDNs received training on use of the GWG chart available in the Epic® EHR. Once enrolled, the participant remained part of the study until her postpartum visit at which point the final survey was emailed or mailed, as per the participant's preference.

### 2.3 Study intervention components

Usual Care participants received written educational materials (developed by Geisinger) regarding GWG and nutrition and counseling by their obstetric care provider. Importantly, referral to a RDN may have been offered as part of UC, independent of randomized assignment.

**FIGURE 1:**
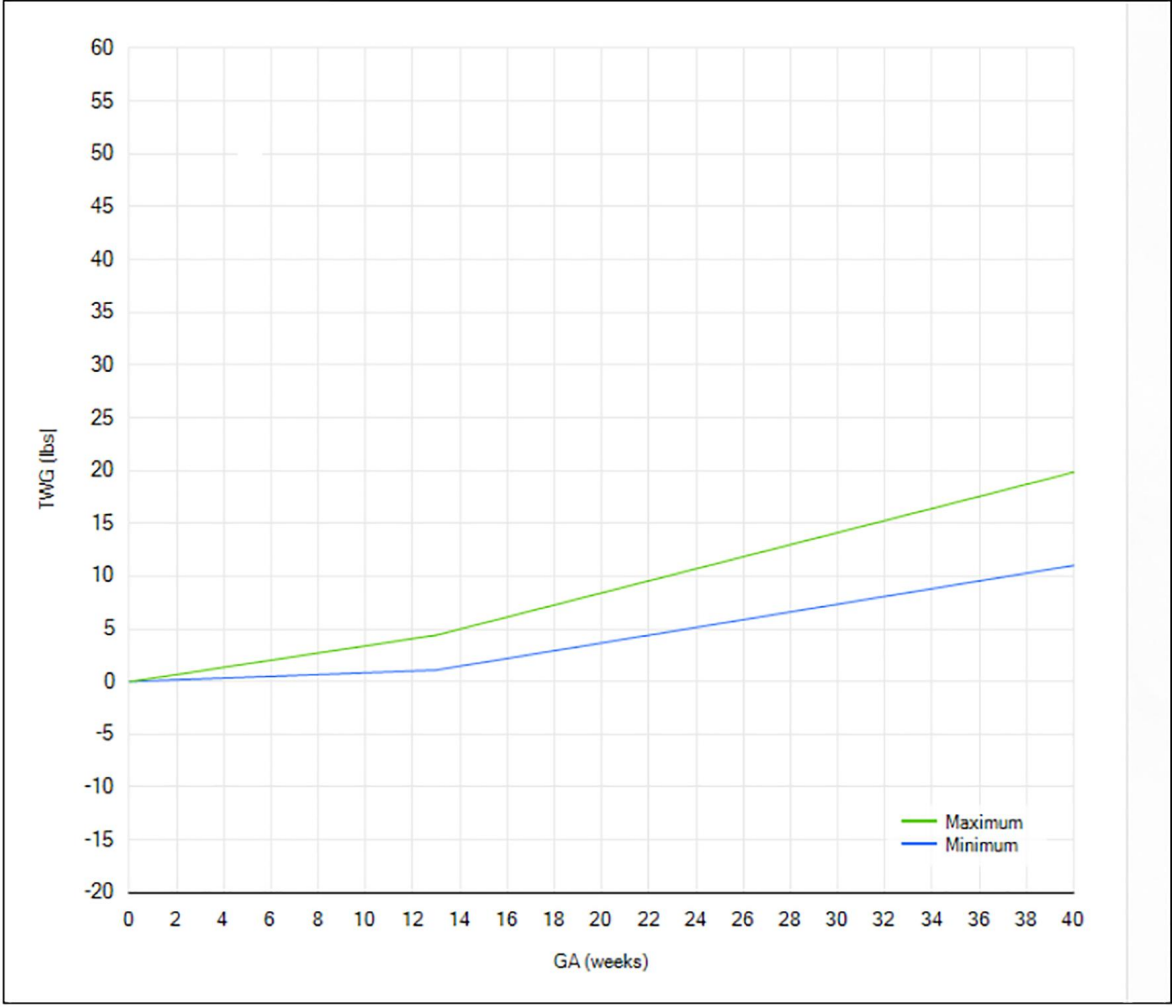
Gestational weight gain chart delineating appropriate gestational weight gain for obese gravida. GA, gestational age; TWG, total weight gain. *Source*: 2021 Epic Systems Corporation

The EC arm offered participants UC plus (a) a personalized letter detailing appropriate GWG (i.e., 5.0–9.1 kg) from the physician principal investigator mailed at enrollment, (b) exposure to a personalized GWG chart in the EHR via the patient portal, and (c) an initial consult and continued counseling with a licensed RDN (10–20 min/checkup every 1–2 weeks) for the duration of the pregnancy. The purpose of the letter from the physician principal investigator was to ensure that all EC participants received GWG information, in case the participant did not receive it (or remember it) during her clinical interaction with the obstetric care provider. Participants who were randomized to the EC arm were encouraged to sign up for *myGeisinger*, the Epic® MyChart patient portal that allows patient access to the EHR. Through the patient portal, EC participants could access a personalized GWG chart at any time (including during telehealth conferences with the RDN). Most (93%) EC participants were enrolled in the *myGeisinger* patient portal. Enhanced Care participants who did not wish to enroll in *myGeisinger* had a GWG chart, with IOM guidelines delineated, mailed to them (Figure 1) and were encouraged to review the chart during conversations with the RDN. The letter and personalized GWG chart components could feasibly be introduced into routine clinical care if desired effects were observed with the EC intervention. Usual Care participants did not have access to personalized GWG charts, to prevent against contamination.

The EC arm included a lifestyle modification intervention delivered by RDNs who used educational strategies to improve participant knowledge and awareness of appropriate GWG and counseling strategies to guide participants in achieving healthy weight behaviors and appropriate GWG. The intervention content was adapted from interventions for gravida with obesity and grounded in social cognitive theory and behavior change principles based on evidence from a prior successful lifestyle/behavior change intervention.^23^, ^25^

Four Geisinger‐employed RDNs who were experienced with prenatal nutrition and behavioral counseling and trained in motivational interviewing interacted with the EC participants to provide counseling that was consistent with standard practice.^21^ For the purpose of this study, RDNs were encouraged to work in partnership with each participant to set and achieve personal nutritional goals for appropriate GWG. As part of typical RDN care, patients were assessed for physical activity levels, and education was provided when warranted. The RDNs encouraged physician approval prior to engaging in strenuous physical activity.

Consultation with the RDN was 45–60 min for the first visit and was conducted in‐person in a Geisinger clinic or by telephone, per participant preference, to optimize convenience and compliance. Telehealth consults were added as an enhancement intended to enrich the focus on weight management by allowing participants continued correspondence with a highly skilled professional and to increase compliance with study visit attendance. Telehealth with RDNs for weight management has been demonstrated to be as efficacious as in‐person consultations, and virtual delivery models reduce travel and time burden.^26^, ^27^ Weekly telephonic nutrition coaching was scheduled for 20 min per check in. This ongoing support is consistent with evidence‐based recommendations for lifestyle modification interventions.^28^, ^29^

Participants were asked to maintain daily food journals, and when these were unavailable, counseling sessions began with 24‐hr recall. Patient‐centered counseling focused on behavior modification, for example, improving nutrient intake (increase fruits/vegetables and decrease refined grain consumption) or reducing high calorie/unhealthy foods that were contributing to rapid weight gain.^8^, ^9^ Registered dietitians/nutritionists guided healthy food and beverage choices and discussed strategies to achieve balance. When necessary, related written educational materials were sent to the participants to further extend learning. As applicable, RDNs discussed ways to utilize healthy foods and budgeting to make food dollars stretch. At the conclusion of each visit, the patient in partnership with the RDN set realistic, measurable goals to help meet GWG targets, for example, choosing 2–3 healthy snacks daily or increasing water intake to greater than 64 ounces/day. Subsequent sessions began with the RDN reviewing patient's prior goals, discussing progress, acknowledging success, troubleshooting challenges, and refining or setting new goals. This process continued until delivery. The messages conveyed by the RDNs during the consult and telehealth visits were not unique to the study, per se; the novelty was that the study made RDN care available to manage GWG and accessible via office visit and/or telehealth. Thus, the approach may be generalizable to RDN consultation in pregnancy and feasible given the rapid adoption of telehealth.^24^

Registered dietitian/nutritionist documentation of care was stored in a study folder for follow‐up sessions and was not shared in EHR in order to avoid contamination. However, as the goal of this study was to *enhance* care for patients, for specific parameters (e.g., pica and food insecurity) that were pre‐agreed upon by the study team and RDNs, the RDN notified the participant's obstetric care provider. Registered dietitians/nutritionists provided counseling for GWG, pica, and food security consistent with standard of practice for dietetics professionals.

### 2.4 Outcome measures

As excessive GWG is associated with further complications in an already higher‐risk pregnancy of a gravida with obesity, the primary outcome was the proportion of participants who gained less than or equal to the maximum amount recommended by the IOM (9.1 kg), that is, the proportion of participants who did not gain excessively; this was adjusted for gestational age at delivery. Gestational weight gain was also assessed as a continuous variable and assessed within categories according to IOM guidelines (i.e., less than IOM [<5.0 kg], within IOM [5.0–9.1 kg], greater than IOM [>9.1 kg]). These parameters were analyzed for all participants in aggregate as well as stratified by prepregnancy obesity class. Logistic regression was used to analyze GWG as a binomial outcome, linear regression for GWG as a continuous variable, and multinomial regression when GWG was defined as three categories. Pre‐specified adjusted analyses of all GWG outcomes were performed and included variables known to be potential confounders: age at delivery, race, parity (nulliparous vs. parous), and insurance (private vs. public). Pre‐specified sensitivity analyses of total GWG were performed for participants with more than six documented prenatal appointments in the EHR and excluding all non‐study–related referrals to a RDN, identified from the participant's EHR. The number of participants that were referred and consulted with a RDN (irrespective of randomization arm) and non‐study indication for referral were assessed. In post hoc analyses, the study evaluated whether there was a difference in GWG per the IOM categories for each class of obesity between participants that were referred versus not referred to a RDN.

Gestational weight gain was assessed by subtracting the prepregnancy weight from the weight at time of delivery. Prepregnancy weight was derived from EHR chart abstraction of weights recorded between 6 months prior to the last menstrual period and 6 weeks 6 days after the last menstrual period. The default reference for the prepregnancy weight was the documented weight closest to 7 weeks prior to the last menstrual period. If missing, patient's self‐reported weight was used. To ensure accurate assessments of patient weight throughout pregnancy, signs were placed on all applicable weighing scales within the individual clinics as a reminder to both patients and staff to remove coats/sweaters/jackets/purses/shoes to allow for more accurate weight assessments. Upon admission to Labor and Delivery, nursing staff obtained the participant's weight. The lead investigator contacted the nursing managers at the two included delivery hospitals to ensure that the staff obstetric nurses were trained on how to appropriately assess patient weight, that is, with minimal clothing on (or in hospital gown) and without shoes.

Pregnancy and neonatal outcomes were compared between groups using clinically derived data obtained from participants' EHRs. In other words, the study team did not set parameters to define or diagnose these clinical outcomes. Pregnancy outcomes included development of diabetes during pregnancy, presence of hypertensive disorders of pregnancy (pre‐existing and pregnancy‐related elevated blood pressures), fetal growth restriction (estimated fetal weight <10th percentile), and mode of delivery (i.e., vaginal). Data abstracted from neonatal EHRs included gestational age at delivery, birthweight, neonatal hypoglycemia (low glucose), respiratory distress syndrome (a breathing disorder that sometimes affects preterm and early term neonates, as diagnosed by the neonatology team), admission to neonatal intensive care unit, and length of stay. Data were abstracted by study personnel blinded to the arm to which the patient was allocated.

Participant self‐reported data were collected by survey at time of enrollment and included demographic characteristics such as age, race, education, insurance, income, marital status, WIC (Special Supplemental Nutrition Program for Women, Infants, and Children) status, parity, and prepregnancy weight and height.

### 2.5 Sample size

A minimum of 100 participants in each clinical arm would yield 80% power to detect a difference between the group proportions of −20% (reduction in proportion of participants that gain more than recommended). Based on past studies of pregnant women with obesity,^14^, ^23^ a withdrawal/drop‐out rate of 25% was applied. Thus, for adequate power to detect a significant difference in the primary outcome, a minimum of 130 participants in each arm were necessary to account for possible withdrawal/drop‐out to achieve the target 100 participants/arm.

### 2.6 Statistical analysis

Continuous variables were expressed as medians and interquartile ranges; frequency and proportions were used for categorical variables. Descriptive analyses were performed to characterize the study groups and to confirm that randomization resulted in no clinically important group differences at baseline. Participants who withdrew were excluded from the final analyses. Participants who experienced miscarriage after randomization or were lost to follow‐up, due to delivery outside of the system, and inability to obtain delivery records including weight, did not contribute data to the analyses of outcomes. Analyses were performed based on intention‐to‐treat as originally planned since the percentage of participants with missing outcome data was <10%, and no differences were detected in baseline characteristics between participants who withdrew and participants who completed; therefore, it is assumed the data were missing due to random, non‐systematic issues. A *p*‐value less than 0.05 was considered significant. Statistical analysis was performed using SAS (version 9.4, SAS Institute Inc.).

## 3 RESULTS

**FIGURE 2:**
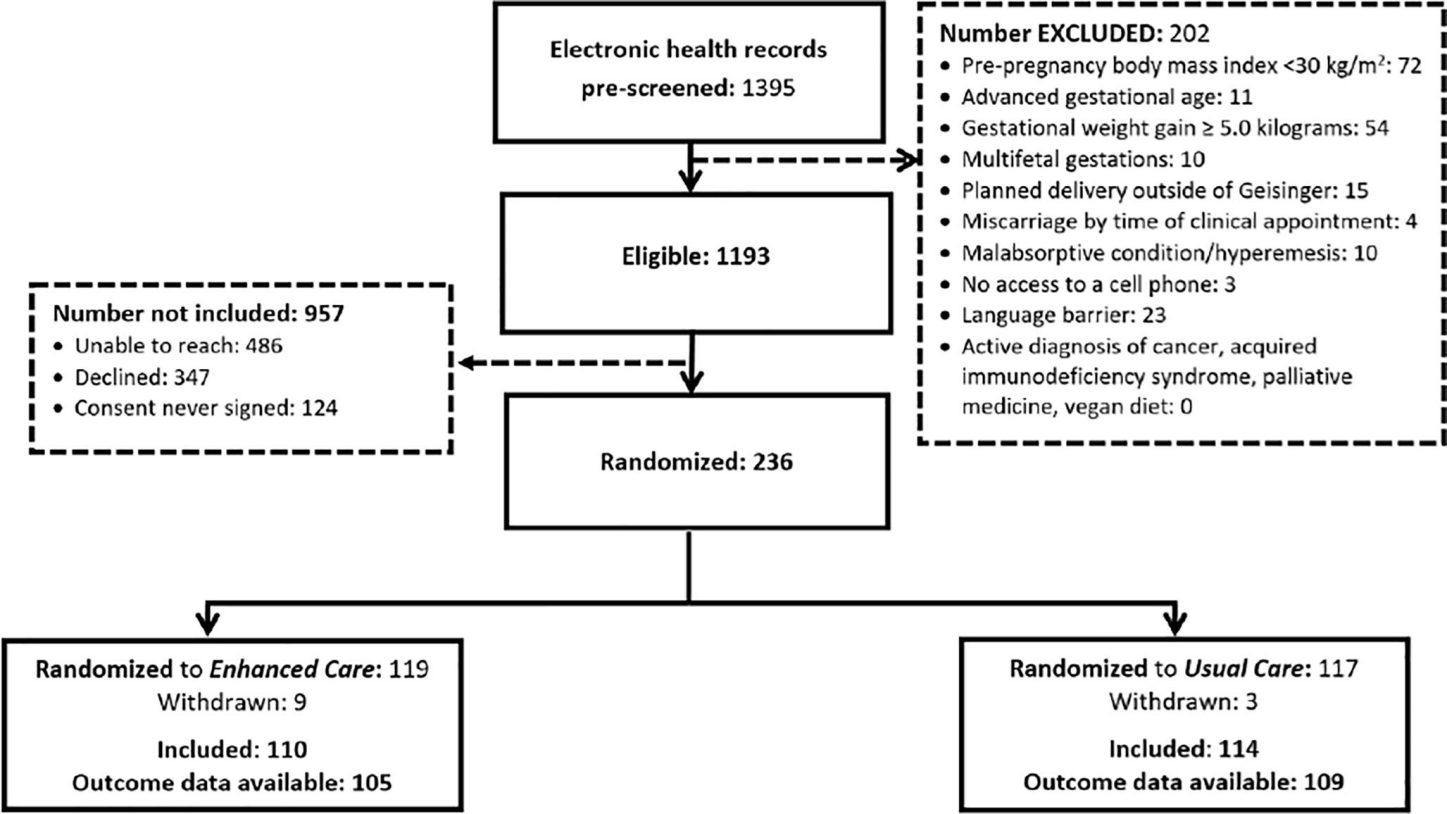
CONSORT statement

Using the EHR, 1395 potentially eligible participants who had appointments scheduled with Obstetric or Maternal‐Fetal Medicine providers within Geisinger were pre‐screened: 202 did not meet inclusion/exclusion criteria. Therefore, 1193 women were eligible: 347 declined, 486 could not be reached, and 124 did not return a signed consent during the trial enrollment period. Therefore, 236 participants with signed consents (33.4% positive response rate) were randomized for inclusion in this trial (see Figure 2 CONSORT statement): 119 to EC and 117 to UC. Nine withdrew from the *EC arm*, four had miscarriages, and one was lost to follow‐up; therefore, outcome data were available for 105 participants in the EC arm. Three withdrew from the *UC arm*, four had miscarriages, and one was lost to follow‐up; therefore, outcome data were available for 109 participants in the UC arm. As there was less loss to follow‐up/dropout than was anticipated, once the study reached the target of at least 100 participants per arm, enrollment ceased.

Demographic characteristics were comparable between the groups. The median maternal age was 29.0 years (interquartile range [IQR] 24.7–32.5) and gestational age at enrollment was 12.1 weeks (10.1–13.9). Of all subjects, 89% were non‐Hispanic white, 49% with at least a college education, 55% privately insured, 28% single, and 29% nulliparous. Approximately 46% of the participants had class I, 26% class II, and 28% class III obesity. Both arms were similar with respect to age at delivery, demographic and socioeconomic characteristics, parity, and prepregnancy BMI (Table 1). Additionally, there were no significant differences in baseline characteristics between participants that withdrew and participants that continued in the trial.

**TABLE 1:** Baseline characteristics of participating patients^a^

Baseline characteristics	Enhanced care	Usual care
	*N* = 110	*N* = 114
Age at start of pregnancy in years, median (IQR)	28.1 (23.7–32.4)	29.4 (25.5–32.6)
Race: Non‐Hispanic white	101 (91.8)	98 (86.0)
Education		
<High school	1 (0.91)	2 (1.8)
High school	28 (25.5)	34 (29.8)
College	40 (36.4)	47 (41.2)
Graduate	13 (11.8)	10 (8.8)
Unknown/Other	28 (25.5)	21 (18.4)
Insurance		
Private	63 (57.3)	60 (52.6)
Public	41 (37.3)	46 (40.4)
Both	4 (3.6)	7 (6.1)
None	2 (1.8)	1 (0.88)
Annual household income		
<$10,000	11 (10.0)	12 (10.5)
$10,000–$24,999	7 (6.4)	17 (14.9)
$25,000–$49,999	20 (18.2)	26 (22.8)
$50,000–$74,999	16 (14.6)	14 (12.3)
$75,000–$99,999	8 (7.3)	14 (12.3)
$100,000–$124,999	5 (4.6)	10 (8.8)
$125,000–$149,999	7 (6.4)	4 (3.5)
≥$150,000	3 (2.7)	2 (1.8)
Do not know/Did not answer	33 (30.0)	15 (13.2)
Marital status: Single	33 (30.0)	30 (26.3)
Enrolled in WIC: Yes (missing 25 [EC]; 15 [UC])	19 (22.4)	34 (34.3)
Parity, median (IQR)	1 (0–2)	1 (0–2)
Parity: Nulliparous	30 (27.3)	34 (29.8)
Prepregnancy BMI class		
Obesity class I (30.0–34.9 kg/m^2^)	49 (44.5)	53 (46.5)
Obesity class II (35.0–39.9 kg/m^2^)	29 (26.4)	30 (26.3)
Obesity class III (≥40.0 kg/m^2^)	32 (29.1)	31 (27.2)
Prepregnancy weight in kg, median (IQR)	96.6 (83.9–111.1)	94.3 (86.2–108.9)
Height in cm, median (IQR)	163.3 (158.1–170.2)	162.6 (160.0–167.6)
Prepregnancy BMI, kg/m^2^, median (IQR)	35.3 (31.8–40.1)	35.4 (32.2–39.8)
Gestational age at enrollment, wks, median (IQR)	12.1 (10.4–13.7)	11.9 (9.7–14.1)

Abbreviations: BMI, body mass index; EC, Enhanced Care; IQR, interquartile range; WIC, Women, Infants and Children Program; UC, Usual Care.

^a^ Excludes withdrawals (9 EC; 3 UC); includes four miscarriages and one lost to follow‐up per arm; data expressed as *n* (%) unless otherwise noted.

As outcome data were not available for 5 participants in each group (secondary to miscarriages and lost to follow‐up), presented outcomes are based on 105 participants in the EC arm and 109 participants in the UC arm. Of the participants randomized to the EC arm, 73 (70%) attended at least the initial consult: The median number of visits was 4 (IQR 2, 9), and median total number of minutes spent in consultation was 99 (IQR 55, 177).

When assessing all included participants, GWG did not significantly differ between study arms regardless of how GWG was defined and even after adjusting for age, race, parity, and insurance. There were no significant differences in the proportion of participants who gained less than or equal to 9.1 kg over the course of pregnancy (40.0% EC vs. 45.0% UC, adjusted *p* = 0.39), total GWG (median of 9.5 kg EC vs. 7.7 kg UC, adjusted *p* = 0.23), and the proportion who gained less than, within or greater than the IOM recommendations (Table 2). There remained no significant differences between the groups for the sensitivity analyses performed: GWG for participants with >6 prenatal obstetric appointments (9.5 kg EC vs. 7.8 kg UC, *p* = 0.20), and when excluding all non‐study indicated RDN consults (9.7 kg EC vs. 8.5 kg UC, *p* = 0.25).

**TABLE 2:** Gestational weight for all patients and then stratified by prepregnancy obesity class^a^

Outcomes	Enhanced care	Usual care	Odds ratio (95% confidence Interval)	*p* value	Odds ratio (95% confidence Interval)^c^	Adjusted *p* value^c^
All patients, *n*	105	109				
GWG ≤9.1 kg^b^	42 (40.0)	49 (45.0)	0.8 (0.5–1.4)	0.39	0.7 (0.4–1.3)	0.30
Total GWG, kg(median, IQR)	9.5 (4.5–13.6)	7.7 (3.9–12.3)	−1.9 (−5.7–1.9)	0.33	−2.3 (−6.1–1.4)	0.23
GWG within IOM^b^	23 (21.9)	28 (25.7)	0.9 (0.5–1.6)	0.62	0.8 (0.4–1.6)	0.55
GWG per IOM^b^, reference group: Within IOM				0.44		0.34
Less than IOM	26 (24.8)	34 (31.2)	1.0 (0.5–2.2)		1.0 (0.5–2.2)	
Greater than IOM	56 (53.3)	48 (44.0)	0.7 (0.4–1.4)		0.7 (0.3–1.3)	
Obese class I, *n*	47	49				
GWG ≤9.1 kg^b^	15 (31.9)	20 (40.8)	0.7 (0.3–1.6)	0.37	0.5 (0.2–1.3)	0.19
Total GWG, kg (median, IQR)	10.9 (7.2–15.2)	9.5 (5.9–13.5)	−3.5 (−8.3–1.2)	0.15	−4.7 (−9.2–−0.19)	0.04
GWG within IOM^b^	10 (21.3)	13 (26.5)	0.7 (0.3–1.9)	0.55	0.6 (0.2–1.6)	0.26
GWG per IOM^b^, reference group: Within IOM				0.67		0.37
Less than IOM	6 (12.8)	8 (16.3)	1.0 (0.3–3.9)		0.8 (0.2–3.4)	
Greater than IOM	31 (66.0)	28 (57.1)	0.7 (0.3–1.8)		0.5 (0.2–1.4)	
Obese class II, *n*	27	30				
GWG ≤9.1 kg^b^	11 (40.7)	16 (53.3)	0.6 (0.2–1.7)	0.34	0.6 (0.2–1.7)	0.32
Total GWG, kg (median, IQR)	9.5 (2.7–13.1)	6.8 (3.2–9.3)	−3.8 (−11.2–3.6)	0.32	−3.3 (−10.9–4.3)	0.39
GWG within IOM^b^	4 (14.8)	12 (40.0)	0.3 (0.07–0.9)	0.04	0.2 (0.06–0.9)	0.04
GWG per IOM^b^, reference group: Within IOM				0.07		0.08
Less than IOM	9 (33.3)	10 (33.3)	0.4 (0.1–1.6)		0.3 (0.08–1.6)	
Greater than IOM	14 (51.9)	8 (26.7)	0.2 (0.05–0.8)		0.2 (0.04–0.8)	
Obese class III, *n*	31	30				
GWG ≤9.1 kg^b^	16 (51.6)	14 (46.7)	1.2 (0.4–3.3)	0.70	1.2 (0.4–3.5)	0.70
Total GWG, kg (median, IQR)	7.0 (3.2–10.0)	4.3 (2.6–14.5)	2.3 (−5.6–10.1)	0.57	1.2 (−6.3–8.7)	0.76
GWG within IOM^b^	9 (29.0)	2 (6.7)	5.7 (1.1–29.3)	0.04	9.4 (1.4–64.3)	0.02
GWG per IOM^b^, reference group: Within IOM				0.10		0.05
Less than IOM	11 (35.5)	16 (53.3)	6.5 (1.2–36.3)		13.2 (1.6–107.3)	
Greater than IOM	11 (35.5)	12 (40.0)	4.9 (0.9–27.9)		7.5 (1.0–55.4)	

*Note*: Greater than IOM, > 9.1 kg; Less than IOM, < 5.0 kg; Within IOM, 5.0–9.1 kg.

Abbreviations: GWG, gestational weight gain; IOM, Institute of Medicine; IQR, interquartile range; kg, kilograms.

^a^ Data expressed as *n* (%) unless otherwise indicated.

^b^ Adjusted for gestational age at delivery.

^c^ Adjusted for age at delivery, race, nulliparous, insurance.

With regards to participants with class I obesity, there were no differences in GWG less than or equal to 9.1 kg or GWG per the IOM categorization. Total GWG was 1.4 kg less in the UC arm, though the GWG in both arms exceeded that recommended by IOM (10.9 kg EC vs. 9.5 kg UC, adjusted *p* = 0.04). With regards to participants with class II obesity, there were no differences in GWG less than or equal to 9.1 kg or total GWG. However, fewer participants gained within the IOM guidelines in the EC arm as compared to the UC arm (14.8% vs. 40.0%, adjusted *p* = 0.04). With regards to participants with class III obesity, there were no differences in GWG less than or equal to 9.1 kg. However, significantly more participants in the EC arm gained within the IOM recommendations as compared to the UC arm (29.0% vs. 6.7%, adjusted *p* = 0.02). Additionally, there was a trend towards significance (*p* = 0.05) favoring the EC arm when comparing GWG less than IOM and greater than IOM to GWG within IOM.

Participants could be referred to and have a consultation with a RDN as part of UC, and this occurred for 36 participants in the EC arm (34.3%) and 45 in the UC arm (41.3%) (*p* = 0.29) for single or multiple indications including gestational diabetes (*n* = 41), obesity (*n* = 33), or other/unknown (*n* = 11). There were neither differences in indication for nor attendance compliance with non‐study RDN consults between arms. The frequency of RDN consults varied by pregestational obesity class. Forty two percent (14/33) of participants with class I obesity in the UC arm versus 19% (5/27) in the EC arm received non‐study RDN consults, and participants with class I obesity had the greatest frequency of completed follow‐up visits (median 3, IQR 0.5–8). Among participants with class II obesity, 5/33 (15%) in the UC arm versus 10/27 (37%) in the EC arm received non‐study RDN consults, but fewer visits were completed (median 1, IQR 0–4). In contrast, 14/33 (42%) participants with class III in the UC arm versus 12/27 (45%) in the EC arm received non‐study RDN consults, and the frequency of completed visits was low (median 1, IQR 0–8). For participants who had any RDN consult (irrespective of randomization arm), an inverse trend was observed between preconception obesity class and the proportion of women whose GWG exceeded the IOM recommendations (60.8% in class I, 52.0% in class II and 35.0% in class III, *p* = 0.02). There were no differences in pregnancy or neonatal outcomes.

## 4 DISCUSSION

The study sought to encourage appropriate GWG in gravida with obesity as they are at the highest risk for excessive GWG‐associated adverse pregnancy outcomes. No significant differences between participants randomized to EC versus UC were observed with regards to GWG less than or equal to 9.1 kg, total GWG, or the proportion who gained less than, within or greater than the IOM recommendations. There were also no significant group differences for pregnancy or neonatal outcomes. When stratified by prepregnancy BMI category, participants with class I obesity randomized to UC gained 1.4 kg less, and participants with class II obesity randomized to UC were more likely to gain within the IOM guidelines as compared to participants randomized to EC. Conversely, participants with class III obesity were significantly more likely to gain within the IOM recommendations when randomized to EC as compared to UC. Additionally, having a RDN consult was associated with a decrease in excessive GWG, particularly for participants with pregestational class III obesity. These findings may be indicative that the extra support by an RDN is specifically helpful for women with class III obesity.

Several studies of interventions designed to enhance appropriate GWG or decrease comorbidities associated with obesity and excessive GWG (e.g., gestational diabetes mellitus) have shown mixed results.^30^, ^31^, ^32^ A Cochrane review of diet and/or exercise for preventing excessive GWG revealed that a combination of these two interventions was likely to decrease excessive GWG. However, in subgroup analysis of women who were overweight or obese and at high‐risk of developing gestational diabetes, these interventions only resulted in a decreased risk of infant macrosomia (i.e., birthweight >4 kg).^33^ In comparison, this study did not reveal a difference in birthweight between the groups.

Similar to this study, a study performed in Australia showed that lifestyle modification interventions did not result in improved GWG in women with obesity.^34^ Another trial targeting women with preconception obesity showed a difference in mean GWG between intervention (brochure or group counseling) and control groups; however, akin to this study, in each group, the majority of subjects gained in excess of the IOM guidelines.^35^ The study populations were similar with respect to age and BMI of participants, however this study had more single and multiparous participants in comparison. As compared to many GWG studies that delivered the intervention separately from clinical care, this study was unique in that the intervention was delivered as an adjunct to clinical care, as the RDNs could view provider notes and had the option to contact providers as needed if concerns about the participant arose.

Though this study did not observe a difference in any of the GWG outcomes when participants were examined in aggregate, some differences were observed when participants were stratified by pregestational obesity class. Enhanced Care appeared to benefit participants with class III obesity, but was not beneficial among participants with class I and class II obesity. This may be related, in part, to exposure of some UC participants to non‐study RDN referrals as any nutrition care may have influenced GWG. The dose of non‐study RDN care among UC participants (42% vs. 19% EC arm) with class I obesity likely confounded the ability to detect differences in GWG outcomes between these sub‐groups. However, non‐study RDN consults likely did not confound the ability to detect differences among participants with class II or III obesity because the frequency of consults was higher in the EC group (class II) or similar between groups (class III), but visit completion rate was low overall.

There are several possible reasons why some studies have demonstrated a significant effect on GWG with intervention while this study did not. While RDN counseling was focused on individualized dietary recommendations, the focus was not on encouraging a diet with a low glycemic index.^5^ A low glycemic index diet may result in a more balanced glycemic environment which has implications for the mother and the fetus.^36^ Trials demonstrating that a low glycemic index diet and diet in combination with physical activity are effective at preventing excessive GWG tend not to be exclusive to women with pregestational obesity.^33^ Women with average weight at preconception tend to gain more weight than women with pregestational obesity, and are in fact, encouraged to do so according to the IOM. Therefore, it stands to reason that a greater difference in GWG may have been noted with inclusion of non‐obese participants and stratification of analyses by prepregnancy BMI class.

Alternatively, a more rigorous nutrition intervention may be warranted. Prior research has demonstrated the efficacy of a more structured meal plan including partial meal replacements with lifestyle modification interventions on appropriate GWG among women who were overweight or obese at conception.^20^ Also a more structured physical activity regimen may be warranted. While RDNs discussed physical activity, they did not track this measure. Patients in this institution may be more likely to receive counseling regarding appropriate GWG, diet, and exercise from their health care provider than would typically be expected^37^; this may have resulted in less difference in GWG between arms.

Finally, almost two fifths of the participants were referred to a RDN, outside of study‐related reasons. This may be reflective of a clinical and/or patient concern about obesity and/or its consequences in pregnancy and possibly related to study‐delivered training to providers on IOM GWG guidelines. The rate of referrals to RDNs in this study was observed using EHR data and was slightly higher than self‐reported rates. In a cross‐sectional survey, 30% of obstetricians report referring women for obesity treatment,^10^ a rate that is at least double the proportion of adults referred for obesity treatment from primary care.^10^, ^38^, ^39^

This study has several strengths. The EC intervention components (including the letter from the physician and patient access to a personalized GWG chart) are feasible to incorporate into routine care. Investigators were responsive to study challenges, including participant's lack of desire for in‐person RDN counseling and slow enrollment. Steps were taken to ensure appropriate assessment of weight at all outpatient and inpatient locations. As having a final weight within 1 week of delivery was imperative for the reliability of GWG results, the study team assessed how often the L&D weight was consistent with the weight at the last clinical appointment: there were 21 EC and 20 UC patients with consistent weights. The median time between the last clinical appointment and delivery was 4 days (with maximum of 7 days). This substantiates that the weight taken when the subject presented for delivery was a true measure of subjects' final gestational weight.

Limitations of the study included the 33% response rate for consenting to participate. However, this is in line with other trials attempting to reduce GWG in gravida with obesity.^20^, ^40^ The majority of subjects were non‐Hispanic white, which limits generalizability of the results to women of other races/ethnicities. Finally, women were referred for RDN consultation for reasons unrelated to the study. However, this was permitted per protocol as the study did not aim to interfere with or prevent usual referral patterns when providers felt RDN consultation was indicated. Rather, the study accounted for these non‐study–related RDN visits through statistical analysis.

Though there were no significant differences in GWG outcomes between the groups, it appears that a letter from a physician outlining appropriate GWG and RDN consultation utilizing access to a personalized GWG chart are helpful for encouraging GWG within IOM guidelines for women with class III obesity, specifically. These interventions did not benefit women with class I or II obesity. Additionally, a RDN consult resulted in a significant decrease in the proportion of women with excessive GWG with an increasing effect as BMI class increased. These data support RDN consultation for pregnant women with class III obesity as a potential way of decreasing the adverse outcomes associated with excessive GWG.

## CONFLICT OF INTEREST

None of the authors on this study have any competing interests.

## AUTHORS CONTRIBUTIONS

Awathif Dhanya Mackeen was the Physician Principal Investigator on this project and developed the protocol and designed the study with assistance from other authors, performed data analysis and interpretation, and wrote the first draft of the manuscript. Lisa Bailey‐Davis, Danielle Symons Downs, and Jennifer S. Savage were Co‐Investigators that assisted with protocol development, study design, survey selection, data analysis and interpretation, and critical editing of the manuscript. Vonda Hetherington is the Director of Clinical Nutrition who assisted with study design, staffing for the RDN visits/phone calls, and critical editing of the manuscript. Shawnee Lutcher was a Research Assistant primarily responsible for patient recruitment and maintaining survey collection for enrolled participants. Jacob W. Mowery was the Project Manager who oversaw project expenses and development of informational technology‐based recruitment strategies and data collection. Amanda J. Young was the biostatistician and assisted with study design; she was responsible for analyzing all data and assisting with data interpretation. All authors read and approved the final manuscript.
